# Toxicity and biodistribution of lanthanum and gadolinium in *Daphnia magna* following chronic dietary and waterborne exposure

**DOI:** 10.1007/s10646-025-03013-4

**Published:** 2026-01-03

**Authors:** Marion L. Revel, Chantal K. E. van Drimmelen, Frederika Černíková, Edith Padilla Suarez, Andrew Hursthouse, Susanne Heise

**Affiliations:** 1 Life Sciences, Hamburg University of Applied Science, Ulmenliet 20, D-21033 Hamburg, Germany; 2 University of the West of Scotland, Paisley, PA1 2BE UK; 3 Institute of Environmental and Chemical Engineering, University of Pardubice, Studentská 573, 532 10 Pardubice, Czech Republic; 4 Biology Department, University of Naples Frederico II, via Cinthia 21, 80126 Napoli, NA Italy; 5 Present Address: Eawag, Swiss Federal Institute of Aquatic Science and Technology, Department of Environmental Toxicology, Dübendorf, Switzerland; 6 Present Address: Hoogheemraadschap Hollands Noorderkwartier, Department of Projects, Advice, and Research, Heerhugowaard, The Netherlands

**Keywords:** Rare earth elements (REE), Chronic exposure, Dietary exposure, Bioaccumulation, Biodistribution, Daphnia magna

## Abstract

*Daphnia magna* has frequently been used to assess the toxicity of lanthanides (Ln) such as lanthanum (La) and gadolinium (Gd). However, most studies have focused on acute toxicity. Knowledge of chronic toxicity and the potential effects of dietary exposure on aquatic organisms is still scarce. The objective of this study was to investigate the effects of chronic waterborne and dietary route exposures of 0.5 mg L^− 1^ of La and Gd on the mortality, growth, and reproduction of *D. magna.* Four different exposure conditions were used: (i) control, (ii) dietary exposure, (iii) waterborne exposure, and (iv) a combination of dietary and waterborne exposure. The results showed that none of the Ln exposures affected the mortality or the growth of the organisms. Reproduction was identified as a more sensitive endpoint. For La, combined waterborne and dietary exposure delayed the release of the first brood by an average of approximately 1.1 days compared to the control group For Gd, dietary and combined exposures significantly decreased the total offspring of the organism by approximately 20 offspring per adult in 21 days. The biodistribution patterns differed for each metal, with La being uniformly localized in the intestine and Gd bioaccumulating differently depending on the exposure route. Dietary and combined Gd exposure led to an accumulation in the intestinal tract. However, waterborne exposure resulted in a more heterogeneous biodistribution within the individual *D. magna*. For both metals, dietary exposure led to the highest Ln body burden in the organisms, in contrast to the waterborne exposures. The effects observed confirm the importance of considering dietary exposure in long-term bioassays. A better understanding of the mode of action of Ln on *D. magna* is needed and could be achieved by exploring the effects of Ln accumulation in the gut and energy limitations for *D. magna*.

**Supplementary Information:**

The online version contains supplementary material available at 10.1007/s10646-025-03013-4.

## Introduction

Lanthanides (Ln) are a group of 15 elements that are increasingly used in society, leading to the release of Ln into the aquatic environment (e.g., through hospital and industrial wastewater) resulting in elevated environmental concentrations (Bau and Dulski 1996). Their natural background concentrations in surface waters remain low (ng L^-1^) but can be found at concentrations of 1 to 10 mg L⁻¹ in stream waters draining areas impacted by Ln mining (Liang et al. 2014; Liu et al. 2019; Revel et al. 2024b). The increasing recognition of Ln as emerging pollutants (Kang and Kang 2020; Revel et al. 2024b) has resulted in a growing number of scientific publications, predominantly addressing their distribution in surface water and sediment, while information on their ecotoxicity remains scarce (Malhotra et al. 2020; Revel et al. 2024b).

Most studies on Ln toxicity have focused on acute effects in aquatic model organisms such as *Daphnia magna* (González et al. 2015; Herrmann et al. 2016; Revel et al. 2023). These studies usually focus on a few representative elements within the Ln group, such as lanthanum (La), the lightest element of the group, which is used in various industrial domains (EC_50_ around 31 mg L^-1^ for *D. magna*), or gadolinium (Gd), a representative medium mass element that is commonly used as a contrast agent for MRI measurements (EC_50_ ranged from 13.93 to 18.5 mg L^-1^ for *D. magna*) (Blinova et al. 2018b; Revel et al. 2023). Most available toxicity data on rare earth elements derive from acute bioassays, which provide limited environmental relevance. Few studies have examined the chronic effects of Ln on *D. magna* (Blinova et al. 2018a, 2021; Shu et al. 2023). At concentrations of La above 0.5 µg L⁻¹, Shu et al. (2023) found that body size was reduced, and the quality and quantity of *D. magna* offspring were concentration-dependent after 21 days of exposure. For Gd, Blinova et al. (2021) reported no toxic effects on *D. magna* after 39 days of exposure to 0.1 mg L⁻¹. These findings suggest that La has a higher chronic toxicity than Gd, which disagrees with the common observation that Ln toxicity increases with atomic number (González et al. 2015; Bergsten-Torralba 2020; Revel et al. 2024a).

The Ln are known for their low solubility, leading to their precipitation and a reduction in the free ion concentration (which is typically considered the bioavailable form) (Vignati et al. 2024). However, metal precipitation challenges the interpretation of toxicity data because crustaceans like *D. magna* can ingest these metal precipitates (Blinova et al. 2018a; Revel et al. 2023). While ingested precipitates may not affect the organism under short-term exposure (Revel et al. 2023), it has been suggested that ingested La and Gd precipitates can trigger hazardous effects under long-term exposure (Revel et al. 2024a. However, the specific impact of dietary exposure, where Ln are taken up through contaminated food rather than as precipitates, remains unexplored. Furthermore, metal ingestion can also occur through the adsorption of metals onto algae, which serve as a food source for daphnids during chronic bioassays.

The potential importance of dietary exposure in chronic toxicity assessments has been demonstrated for other metals such as cadmium (Geffard et al. 2008), nickel (Evens et al. 2011) and arsenic (Wang et al. 2018). For instance, the role of dietary exposure has been highlighted in the chronic toxicity of copper (Cu) and zinc (Zn) to *D. magna* and waterborne exposure has been suggested to be the most toxic route (De Schamphelaere and Janssen 2004; De Schamphelaere et al. (2004). Therefore, the objective of our study was to follow a similar method to explore the role of chronic dietary exposure to two Ln, lanthanum (La) and gadolinium (Gd), on *D. magna.* For that purpose, the method was slighty adapted and three different exposure types were compared for 21 days: dietary (i.e., ingestion of Ln that have been adsorbed to the surface of microalgae), waterborne, and a combination of both. Since the no-effect concentration was predicted to be 0.6 mg L⁻¹ for La and ranging from 0.1 to 1 mg L⁻¹ for Gd (Revel et al. 2024a, 2025), and because Blinova et al. (2018a) did not observe any effects at 0.1 mg L⁻¹ of Gd over 39 days, the present study increased the nominal concentration to 0.5 mg L⁻¹ for both metals. The hypothesis formulated for this experiment was that chronic dietary exposure may affect *D. magna*, but waterborne exposure was expected to be the primary route of toxicity. In addition, a difference in toxicity between La and Gd was expected.

## Materials and methods

### Microalgae preparation

*Raphidocelis subcapitata (SAG Strain Number*: 61.81) was used as a food source for Ln to establish the chronic exposure of *D. magna* to Ln, as suggested by Revel et al. (2024a). This algal species was obtained from the Culture Collection of Algae at the University of Göttingen (SAG), Germany. Microalgae were maintained at the Hamburg University of Applied Sciences (HAW) at 20 ± 1 °C, with lighting intensity between 6000 lx and 10 000 lx (Osram) and an 8:16 light-dark cycle. A total of 4 × 10^5^ cells mL^− 1^ of *R. subcapitata* were incubated for 72 h in ISO media (ISO 2012a) without Ln (control) and with 0.5 mg L^− 1^ of La (III) or Gd(III) (PEN9300127 (La) and PEN9300118 (Gd), calibration standards, Perkin Elmer). The media were prepared in PET bottles to prevent metal precipitation and adsorption to the container walls, and the pH was adjusted to 6.8 (± 0.1) with NaOH (6777.1, Roth) or HCl (836, Geyer). The media were stored at 20 °C in the dark for 48 h before inoculation to allow the solution to reach equilibrium. After the waiting period, the algae were inoculated by carefully adding them to the media at a density of 4 × 10^5^ cells mL^− 1^. Although the inoculation could slightly dilute the Ln concentration, the effect was considered negligible due to the small inoculum volume. At the end of the 72 h exposure, microalgae were harvested by centrifuging at 10 000 g for 10 min using a Thermo Heraeus Multifuge 1 S-R Chilled Centrifuge with TTH-400 Rotor 4 × 400 (Thermo Fisher Scientific, Waltham, MA, USA). The pellet was resuspended in metal-free ISO media to a concentration of 8 × 10^7^ cells mL^− 1^. The algae dilution was stored in the dark at 4 °C and used as food in the 21-day daphnid chronic assay. Every week, a sample of the culture was visually inspected under a microscope to ensure that no significant cell lysis or bacterial growth had occurred during storage.

### Chronic toxicity of Ln on daphnids

The *D. magna* culture was maintained at HAW for several years and was originally acquired from the German Environmental Ministry (Umweltbundesamt). For maintenance, organisms were fed daily with the freshwater algae *Chlorella vulgaris* (Collection of Algae at the University of Göttingen (SAG 211-11b) in Elendt M4 media at a temperature of 20 ± 1 °C with a 16:8 light-dark cycle. *D. magna* reproduces primarily via parthenogenesis under laboratory conditions, producing genetically identical female offspring without sexual reproduction (Ebert 2005). Therefore, sex discrimination or analysis was not applicable in this study, as all individuals were female clones.

The daphnid test medium was prepared from M4-medium (pH 6.8 (± 0.2), according to ISO 6341:2012 (ISO 2012b). However, the EDTA-complex was omitted, as it might interfere with Ln in the test medium. Three M4-media were prepared, with two of them spiked with 0.5 mg L^-1^ of La (III) or Gd (III) in PET bottles. Media were stored for four days in a dark room at 4 °C before being used in order to reach the chemical equilibrium, as described by De Schamphelaere and Janssen (2004). One day before the experiment, the media were stored at 21 °C to avoid high temperature differences for the test organisms.

The 21-day exposure started with neonates younger than 24 h old. Exposure was performed under four different test conditions (see Fig. S1 for a visual representation of the exposures): (i) exposure to metal-free media and fed with non-contaminated algae (control); (ii) dietary exposure that used contaminated microalgae as food but without spiked fluid medium; (iii) waterborne exposure, standard exposure in chronic toxicity test, with *D. magna* exposed to spiked medium (0.5 mg Ln^3+^ L^-1^) and fed with metal-free algae; and (iv) a combined exposure to Ln-spiked media and Ln-pre-exposed microalgae. This experimentation was repeated three times in order to obtain three technical replicates.

For each condition, 10 neonates (e.g., biological replicates) were placed in separate PET cups containing 50 mL of the respective medium. All cups were fed with a specific amount of *R. subcapitata* cells per day per organism, depending on the day of the test (Table 1), to match the increasing nutritional needs of Daphnia as they age (De Schamphelaere and Janssen 2004). An additional cup containing only algae and no daphnid was added for each test condition. This additional cup was used to determine the development of the algal cell concentration without any feeding pressure, from which to calculate the feeding rate.

**Table 1:** Amount of R. subcapitata in cell per day per organism fed over the duration of 21-days

Days	Cells per day per organism
0–6	8 × 10^6^
7–8	1.2 × 10^7^
9–20	1.6 × 10^7^

On days 2, 5, 7, 9, 12, 14, 16, and 19, the microalgae concentration in each cup was determined using fluorescence measurements. Daphnids were then transferred with small volumes of their exposure medium to small beakers, the cups were cleaned with pure water, and the 50 mL of exposure media was renewed using solutions prepared under the same conditions as previously described. The daphnids were then re-introduced. Mortality and offspring of individual *D. magna* were recorded daily. Reproduction was studied with two endpoints: the date of the first brood release and the total number of offspring.

After 21 days of exposure, all adults per condition were collected and immersed in non-spiked M4-medium for 10 min followed by a 5 mM Na2-EDTA (EDTA disodium salt dihydrate, 6381-92-6, Carl Roth) solution for 20 min. The daphnids were dried for 48 h at 40 °C, individually weighed and photographed using a Motic Images Plus 3.0 camera mounted on a Wiloskop stereomicroscope (Hund Wetzlar, Wetzlar, Germany). Subsequently, FIJI software (Schneider et al. 2012) was used to measure the length of each daphnid from the eye to the base of the tail, using the images obtained from the camera.

### Biodistribution of La and Gd in daphnids

Dried *Daphnia* samples were adhered on double-sided tape to the edge of glass slices to study metal biodistribution using micro-X-ray fluorescence (µXRF). The drying and fixation process could cause structural damage to the samples, making analysis difficult; as a result, only 3 to 4 organisms per condition were successfully measured. These measurements were performed at the Helmholtz-Zentrum Hereon’s institute for metallic biomaterials with an M4 Tornado (Bruker), using the following conditions for the area map scans: energy of 50 kV, an anode current of 600 µA, a spot size of 25 μm, and an integration time of 1.5 s pixel^-1^ (Helmholz et al. 2019). One picture was produced per element, showing its distribution on the scanned zone, and was analyzed using the image processing open-access software FIJI (Schindelin et al. 2012). To minimize the background noise as much as possible, the average of the background was calculated, and the minimum display value of the image was adjusted to that average. The different images of interest were then combined and colored.

### Ln body burden of daphnids and microalgae

The metal body burden of *D. magna* and microalgae was quantified through total reflection X-ray fluorescence spectrometry (TRXF; S4 T-Star; Bruker Nano GmbH, Berlin Germany), which requires less sample volume than for example, ICP-MS. The measurements were performed with an anode voltage of 50 kV, an anode current of 1000 µA, energy resolution < 145 eV, and an integration time of 1000 s, using gallium as the internal standard. The microalgae pellets used as food for daphnid were collected after centrifugation and freeze-dried for 72 h under vacuum (0.22 mbar) using a single-chamber ice condenser Alpha 1–4 LSCplus (Martin Christ, Germany).

The dried *D. magna* and the freeze-dried microalgae were digested in 1.5 mL microtubes containing 45 µL of pure water, 400 µL of HNO_3_ (65%, Roth, 4989.1), 50 µL of HCl (35–38%, p.a., Th. Geyer, 836.2500), and 5 µL of gallium (Ga) internal standard (VHG; VHG-PGANH), with a final concentration of 10 µg mL^-1^. After a digestion period of at least 48 h, 20 µl of the digested sample was diluted in 80 µl of pure water and 5 µl of 3% polyvinylalcohol (9002-89-5, Carl Roth). From this dilution, 10µL of solution was pipetted onto a quartz carrier disc and dried at 60 °C on a heating plate. This process was repeated once more before placing the discs in an oven at 80 °C for one hour, before analyzing them with the TRXF. Metal concentrations were calculated from the spectrum obtained and analysed with TEsprit 1.0 software (Bruker Nano GmbH, Berlin, Germany).

### Determination of dissolved metal concentrations

The influence of algal cells used as food on the dissolved Ln concentration was also studied because adsorption or leaching phenomena can cause variations in Ln levels. All exposure conditions were prepared as previously described, but no daphnids were added to the plastic cup. However, microalgae were added every day for 2 days of exposure. An aliquot of 20 mL was filtered through a 0.2 μm cellulose nitrate filter (7182-004, Whatman), and the filtrates were acidified with 0.2% HNO_3_ 63%. The dissolved Ln concentration was then measured using inductively coupled plasma mass spectrometry (ICP-MS) at the Department of Environmental and Chemical Engineering at the University of Pardubice, Czech Republic.

The following reagents were used for the ICP-MS analysis: single-element standard solutions 1.000 ± 0.002 g L^− 1^ of Gd and La (SCP Science, Canada) for instrument calibration, 1.000 ± 0.002 g L^− 1^ of In (Analytika, Czech Republic) as internal standard (all analytical-reagent grade), and ultrapure water (conductivity 0.07 µS cm^-1^, Evoqua Water Technologies, Germany). The calibration solutions contained 0.01, 0.05, 0.1, 0.5, 1, 5 and 10 µg L^-1^ Gd and La. Internal standard (1 µg L^-1^) was added to all samples (blank, calibration standards and samples) which were then diluted 100 times.

An inductively coupled plasma time-of-flight mass spectrometer, OptiMass 9500 (oaTOF-ICP-MS) (GBC Scientific Equipment, Australia), was used to conduct a trace analysis of Gd and La in the samples. The ICP-MS analysis parameters were: plasma power 1200 W; plasma, auxiliary and nebulizer gas flow rates, 12 L min^− 1^, 0.55 L min^− 1^, 0.91 L min^− 1^; multiplier gain 2700 V, acquisition time 5 s; and three replicates. Instrument detection limits were 4.0 × 10^− 7^ mg L^-1^ for Gd and 2.0 × 10^− 7^ mg L^-1^ for La. The quantification was performed using external calibration with an internal standard. Unwanted ranges of m/z (1–112, 118–135, 142–152, and 161–260) were excluded from detection using SMARTGATE ion blanking. The working isotopes were ^139^La and ^158^Gd. They were chosen with respect to possible isobaric overlaps of interfering ions of the same mass using the spectral library of the device and the mass spectrum of the samples.

### Statistical analysis

Statistical analysis (see S2) was performed using GraphPad Prism version 9.3.1 for Windows and data visualizations were created using RStudio (R version 4.3.2).

None of the studied endpoints followed a normal distribution according to the Kolmogorov–Smirnov test (p-value > 0.05). Therefore, dry weight, length, date of release, and number of offspring produced per adults were compared with the control group using multiple Mann–Whitney tests. For these tests, p-values were corrected to control the false discovery rate (FDR). The FDR approach adjusted the p-values using the Benjamini et al. (2006) two-step step-up method to ensure that the proportion of false positives did not exceed the pre-specified threshold (Q = 1%), thereby increasing the reliability of the results.

Kruskal–Wallis tests were used to study the body burden in daphnids and algae in order to compare the four types of exposure with each other. To compare the feeding rate of the daphnids in the different exposure groups, the slope of the linear curve was examined. The 95% confidence intervals (CIs) for the slope were calculated to assess the differences in feeding rates between the exposure groups.

## Results

### Validation of the exposure conditions

**Fig. 1:**
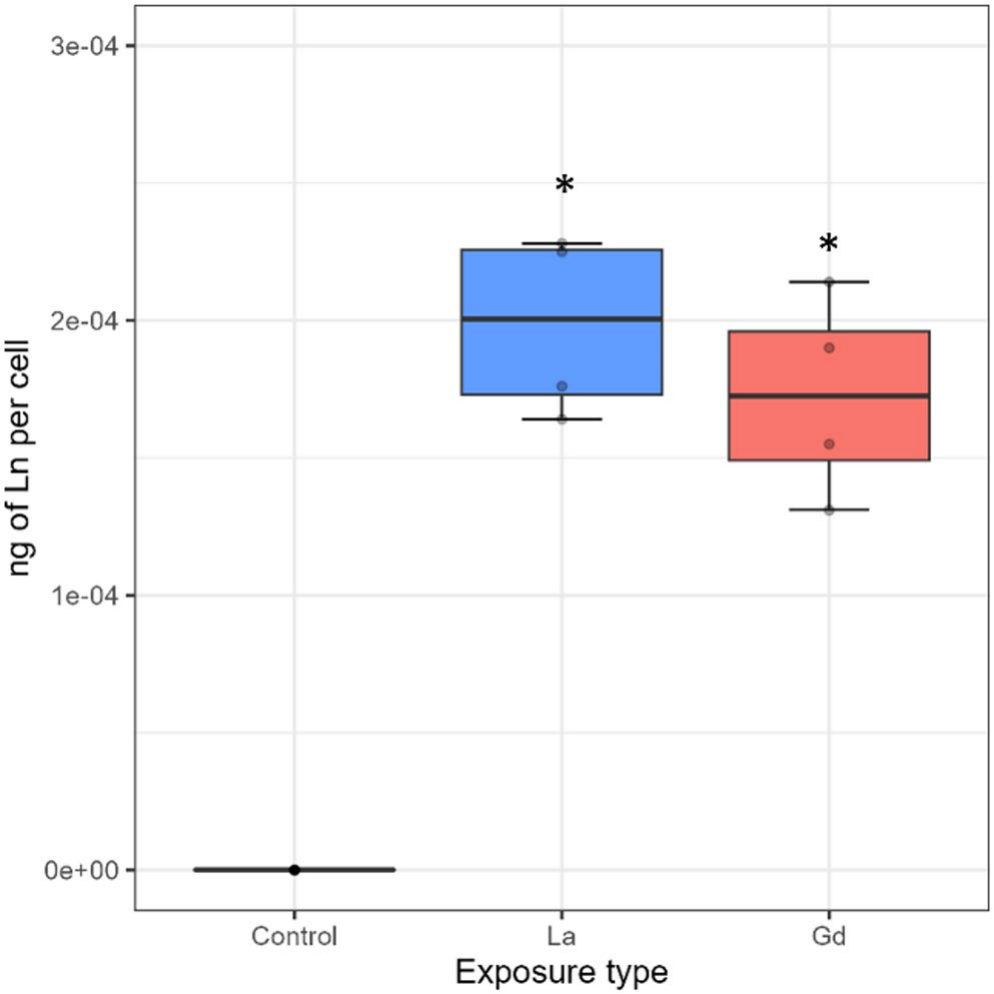
Ln accumulated per R. subcapitata cell (in ng per cell) after 72 h of exposure to 0.5 mg L-1 of La or Gd. Boxplots present the median and the interquartile range (IQR). The whiskers represent the range of that data within 1.5 x IQR from the quartiles. Any points falling beyond that range are considered outliers. Points with the same value are overlapped; therefore, not all points may be visible. Stars indicate significant differences between the control and the Ln exposures with p-values < 0.05

A number of pre-experiments were carried out in order to test the exposure conditions:

- Provision of equal La and Gd concentrations for food-borne exposures: La and Gd exposure (72 h) led to a significant increase in Ln concentration per algal cell compared to the negative controls. Seventy-two hours of exposure to the respective Ln solution led to similar accumulated masses per microalgal cell (2 × 10^− 04^ ng La; 1.7 × 10^− 04^ ng Gd; Fig. 1).
- Influence of algae cells on the dissolved (waterborne) Ln concentration: This was achieved by quantifying the La and Gd concentrations in the different exposure media after three days of exposure across the three different amounts of microalgae provided during the experimentation (Fig. S3). The results indicated that La and Gd exhibited different dissolved concentrations despite having similar nominal concentrations. Specifically, the dissolved La concentrations were much lower than the nominal concentration of 0.5 mg L^-1^, likely due to metal precipitation or adsorption on the cells. Unfortunately, the latter could not be verified because the dry mass of microalgae collected from the *Daphnia* assay was insufficient to accurately determine their internal Ln concentrations.

### Bioaccumulation of La and Gd in daphnids after chronic exposure

**Fig. 2:**
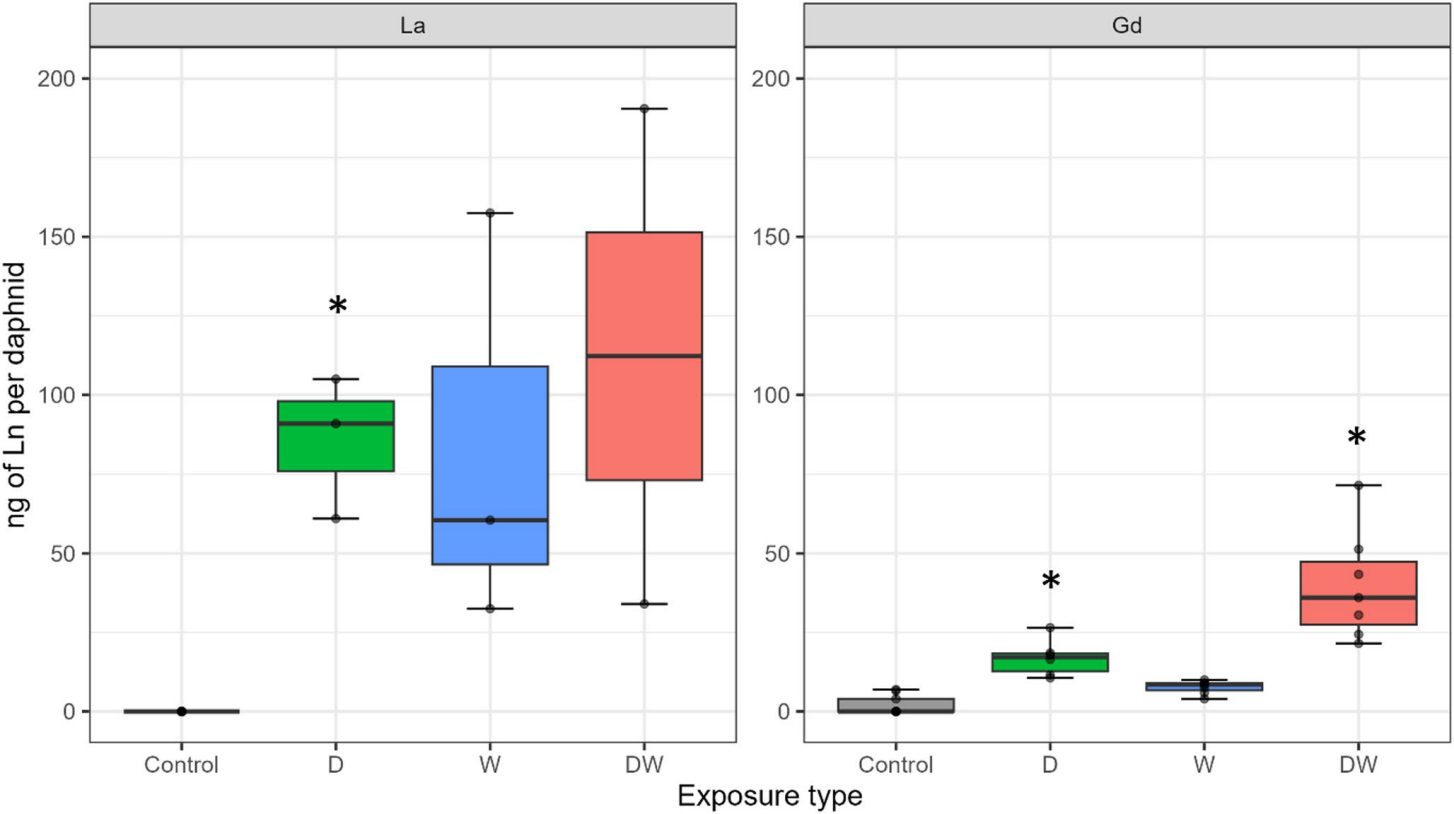
Ln content in D. magna after 21 days of exposure. Boxplots present the median and the interquartile range (IQR). The whiskers represent the range of that data within 1.5 × IQR from the quartiles. Any points falling beyond that range are considered outliers. Points with the same value are overlapped; therefore, not all points may be visible. White: control (Ctr), green: dietary exposure (D), blue: waterborne exposure (W), red: combined exposure (DW). Stars indicate significant differences between the control and the Ln exposures with p-values < 0.05

After 21 days of exposure, the Ln mass in daphnids was measured using TRXF (see Fig. 2). La accumulated more in the daphnids (up to 200 ng daphnid^-1^) compared to Gd (below 80 ng daphnid^-1^) under all exposure conditions. For both metals, waterborne exposure did not differ from the control, whereas dietary exposure led to a significant increase (p-value of 0.02 and 0.01 for La and Gd respectively) in Ln content in daphnids. Combined exposure also resulted in a higher Ln body burden compared to the control (significant for Gd). Due to the higher variability among replicates for La for waterborne and combined exposure, statistical differences for these experiments could not be determined.

**Fig. 3:**
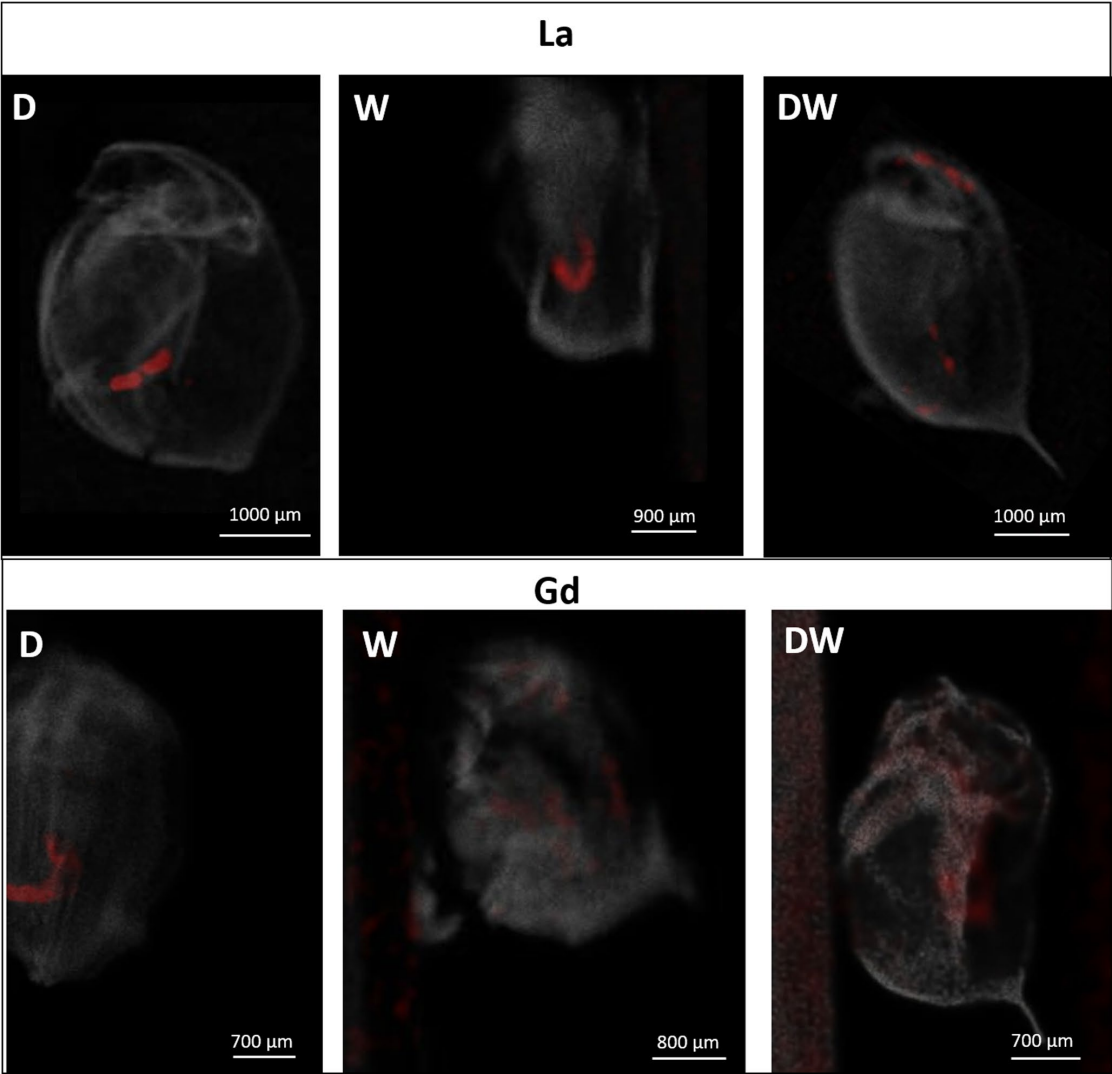
Lanthanide distribution in 21-day-old D. magna according to XRF measurements. Gray: calcium, red: lanthanide. D: dietary exposure, W: waterborne exposure, DW: dietary and waterborne exposure. Energy: 50 kV, pixel size: 25 μm, acquisition time: 1.5 s pixel^− 1^

To collect further information on the toxicokinetic behavior of La and Gd, their biodistribution was studied in the organisms by µXRF measurements (Fig. 3). For reference, the distribution of calcium (Ca) is shown in gray, and the distribution of both Ln is shown in red. For the three La exposures, La appeared to be uniformly localized in the intestine, while for Gd, bioaccumulation differed depending on the type of exposure: dietary exposure led to an accumulation in the intestinal tract, while the waterborne exposure displayed a much more irregular biodistribution within the organism. After combined exposure, Gd was also diffusely distributed in the organism and in the intestinal tract.

### Toxicity in the chronic daphnid test

**Fig. 4:**
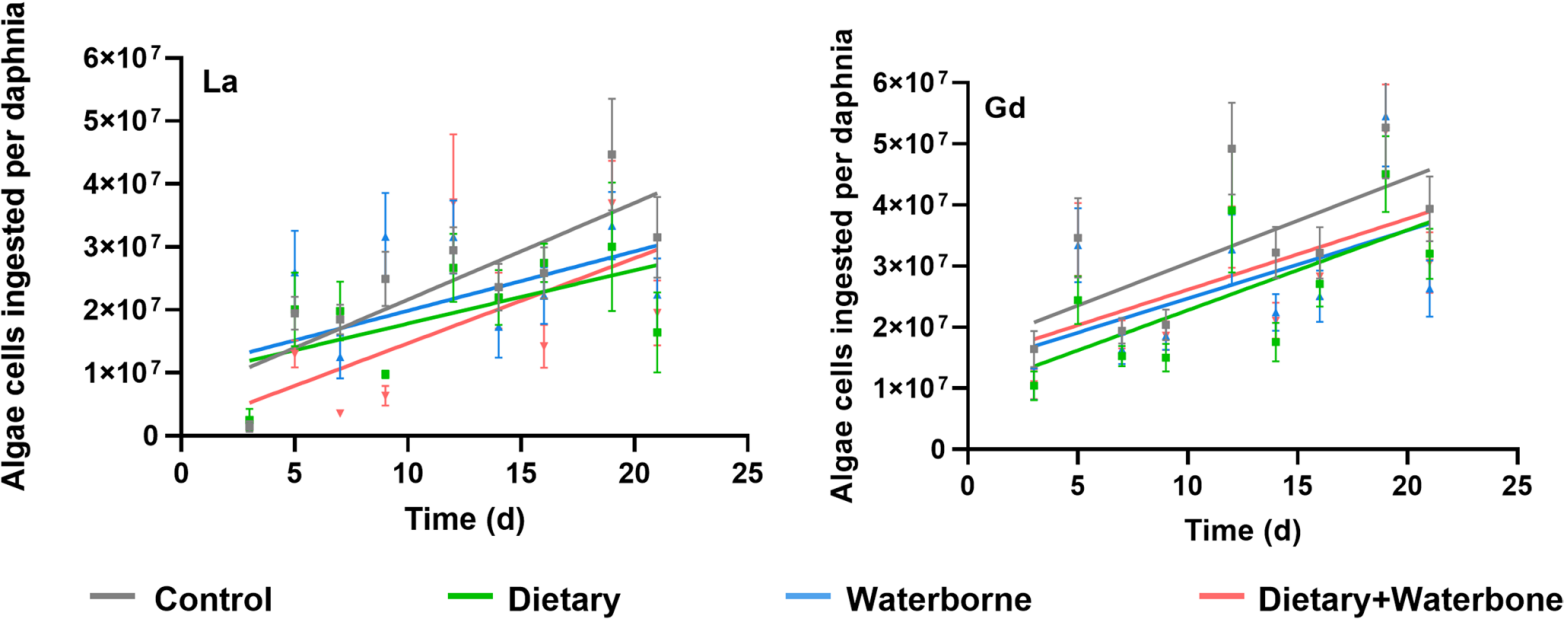
Feeding rate (algae consumption over time) of the organisms exposed to La (right) and Gd (left). Thirty data points per condition and time. Points represent mean values, and error bars indicate the standard error of mean. Equations of each linear curve are available in Table S4

**Fig. 5:**
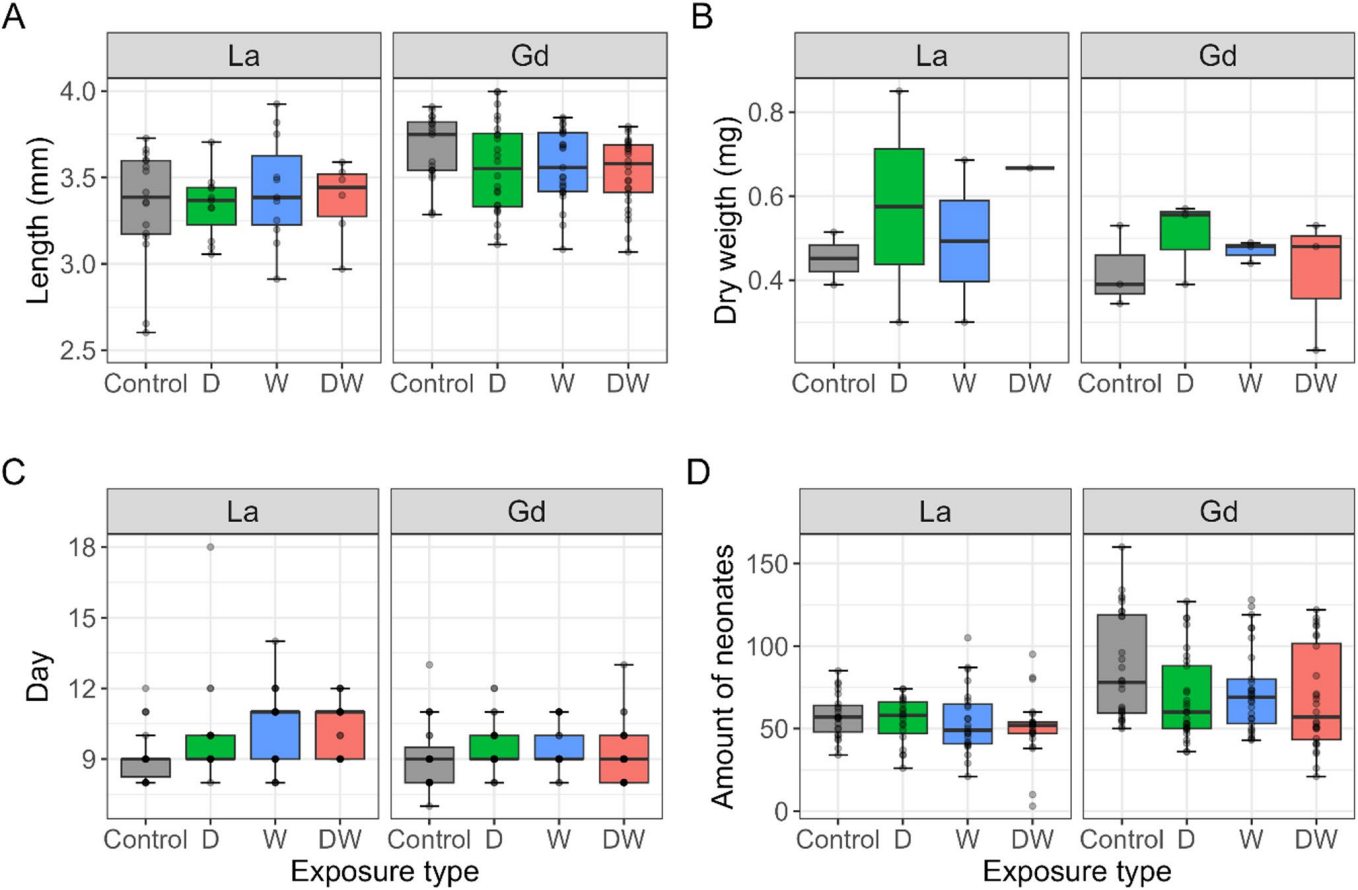
Length **A**, dry weight **B**, day of the first brood release **C**, and total neonates produced per daphnids **D** calculated after 21 days of exposure to 0.5 mg L-1 of La and Gd. Boxplots present the median and the interquartile range (IQR). The whiskers represent the range of that data within 1.5 × IQR from the quartiles. Any points falling beyond that range are considered outliers. Points with the same value are overlapped; therefore, not all points may be visible. White: control (Ctr), green: dietary exposure (D), blue: waterborne exposure (W), red: combined exposure (DW). Stars indicate significant differences between the control and the Ln exposures with p-values < 0.05

After 21 days of exposure, no mortality was found in any of the test conditions regardless of the exposure type. Although a decrease in the feeding rate was observed in daphnids exposed to Ln compared to the controls (Fig. 4), the wide standard error of the mean limited the identification of a significant effect of La and Gd on this endpoint. Similarly, no differences were detected in daphnid length (Fig. 5A), which averaged 3.51 ± 0.26 mm across all conditions, or in dry weight (Fig. 5B), which remained constant at 0.43 ± 0.08 mg for both exposed and control groups. Overall, these findings indicate that La and Gd exposure did not have a significant impact on daphnid mortality, feeding rate, length, or dry weight.

To study the effects of La and Gd on the reproduction of *D. magna*, two endpoints were used: the day of the first brood release (Fig. 5C) and the total amount of neonates produced per daphnid over 21 days (Fig. 5D). Gd showed no significant effects on these endpoints. However, high variability in the control group’s offspring limited statistical power. La exposure, on the other hand, appeared to delay the release of the first brood for daphnids under combined exposure conditions (both waterborne and dietary), by 1.13 ± 1.26 days. Despite this delay, La did not affect the total amount of neonates produced after 21 days.

## Discussion

This study highlights the role of chronic dietary exposure to Ln on *D. magna* reproduction but does not support the hypothesis that waterborne exposure is the primary route of toxicity. The observed effects on reproduction were limited to dietary and combined exposures. No significant effects on survival, growth or dry weight were found. In addition, a negative trend in food consumption was observed, although this was not statistically significant. Despite the large number of data points (30 organisms per day and conditions), considerable variability in feeding rate was observed among individuals. This inter-individual variability likely contributed to the large standard errors and may have reduced the statistical power of the analysis, making it difficult to detect significant differences even when biological effects may have been present.

These results were consistent with several studies showing that dietary metal exposure does not impair overall survival or growth in crustaceans but specifically inhibits their reproduction (Hook and Fisher 2001; De Schamphelaere et al. 2007). However, the absence of discernible effects from the waterborne exposures does not agree with the finding of Shu et al. (2023) that no offpring could be produced by *D. magna* when exposed to waterborne concentrations greater than 0.35 mg L^− 1^. The difference in results between our study and that of Shu et al. (2023) could potentially be attributed to differences in exposure conditions. In particular, the composition of the culture media used could affect metal speciation and bioavailability. However, as the specific details of the media composition in Shu et al. (2023) are not available, this hypothesis cannot be verified. It is also possible that the *D. magna* population used by Shu et al. (2023) was more sensitive to Ln than those used in other studies, including Blinova et al. (2018a), who reported no toxicity effect on daphnids exposed to 0.1 mg L^-1^ of Gd for 39 days.

We show no dependence of body burden or toxicity on the concentration of dissolved Ln. The concentration of dissolved La in the exposure media is consistently lower than for Gd across all exposure types. Reduced concentrations of dissolved La could be caused by stronger biosorption to algae surfaces or by enhanced precipitation within the media. Microalgae have been shown to efficiently adsorb Ln due to the presence of negatively charged functional groups on their cell walls and in their extracellular matrix (Song et al. 2024). Adsorption kinetics of Ln have been assumed to be similar for many surfaces due to the similarity of their hydrated ionic radii and the same, trivalent valence (Li et al. 2015). To the authors’ knowledge, experimental data for *R. subcapitata* do not exist, so this cannot be ruled out. However, the tendency to form precipitates, especially in the form of carbonate complexes, is stronger for La than for Gd, rendering this mechanism the probable cause for lower concentrations (Revel et al. 2023). This results in the formation of particulate matter that can be ingested by the exposed *D. magna* (Revel et al. 2023), explaining the observed biodistribution of the metal in the gut (Fig. 3).

While toxicity did not appear to depend on the concentration of dissolved Ln, the effects on *D. magna* reproduction seemed to be linked to body burden. The highest body burdens were observed in daphnids exposed to dietary and combined exposures (Fig. 2), suggesting that ingestion of contaminated microalgae led to greater bioaccumulation, specifically in the gut (Fig. 3). Such bioaccumulation was found to remain in the gut even after the depuration process (Revel et al. 2024a), suggesting that the elimination of La through faeces may be slower than is typically observed for ingested metals. In contrast, waterborne exposure to Gd resulted in a more widespread distribution throughout the organism which may reflect its greater ability to be absorbed by multiple tissues. Nevertheless, despite this apparent uptake, Gd did not induce sublethal effects at the tested concentrations, suggesting that internal concentrations remained below the threshold for effects.

However, due to high background noise introduced by the use of tape for sample fixation, it was not possible to quantify the Ln signal (e.g., pixel intensity profiles) from the µXRF images. For this reason, body burden was measured separately (Fig. 4) to provide more robust quantitative data. The µXRF imaging was primarily used to gain a general overview of elemental biodistribution, rather than precise quantification. Furthermore, as only 3–4 individuals per group could be successfully imaged due to sample integrity issues, any observed patterns should be interpreted cautiously, as they are qualitative and cannot be statistically tested. Another limitation in this method is its detection limit (usually in the range of µg g^-1^) because it may not allow for the visualization of very low signals compared to other imaging techniques, such as synchrotron XRF (Dias et al. 2015). Nevertheless, we consider µXRF to be a relevant and promising technique for studying metal distribution in daphnids, primarily thanks to its faster analysis capacity. However, improvements in the sample preparation will be necessary to reduce background noise and enable signal quantification in future applications.

In our study, the detection limit may have limited the detection of lower accumulation levels, particularly in eggs, as observed for other metals affecting *D. magna* reproduction (Evens et al. 2012). However, given the strong signals measured in the gut, this is unlikely to affect the main conclusions of the study. Therefore, the result suggests that dissolved forms of Ln, commonly considered the most bioavailable for aquatic organisms (Adams et al. 2020), are not the only chemical species capable of affecting the organisms. This conclusion agrees with Revel et al. (2024a), who recommended addressing both dissolved and particulate forms of Ln for chronic risk assessment.

The bioaccumulation of La and Gd in *D. magna* demonstrates that dietary exposure results in tissue uptake. In the case of La, this goes along with a significant delay in the first brood. Similar effects have been observed with other metals; for example, Evens et al. (2012) showed that zinc (Zn) affected the reproduction of *D. magna* when accumulating in the gut epithelium. Once in the gut, metals can potentially desorb due to lowered pH (around 5.5-6.0) in the gut of daphnids (Davis et al. 2020) and directly affect the gut epithelium. Metals can also inhibit digestive enzyme activity, potentially affecting nutrient absorption (Evens et al. 2012). This hypothesis aligns with the conclusions of De Schamphelaere et al. (2007), who showed that chronic Cu toxicity was not dependent on the dissolved Cu concentration and that dietary exposure could directly affect the reproduction and growth of *D. magna* by inhibiting the vitellogenesis or affecting energy acquisition and consumption.

Although this study highlights the importance of dietary exposures to La and Gd in chronic assays with *D. magna*, it provides limited insight into their relative toxicities. The results suggest two distinct effects on daphnid reproduction: La exposure delayed the first brood, whereas Gd exposure reduced the overall number of offspring produced. These results do not allow us to determine whether one metal is more toxic than the other. The impact of Gd may be more environmentally relevant than the impact of La for *D. magna* reproduction and population dynamics. However, the environmental relevance of these effects, such as a one-day delay or slightly reduced fecundity, should be interpreted with caution. Their translation into population-level impacts would require further validation through dedicated modelling approaches. Furthermore, the exposure concentration of 0.5 mg L^-1^ remain much higher than the average concentration of La and Gd found in surface water (measured in ng L^-1^) (Revel et al. 2024b). However, these effects could be found in stream waters from draining areas that are impacted by Ln mining, where concentrations can rise to 10 mg L^-1^ (Liu et al. 2019).

## Conclusion

We have shown an important role of dietary exposure in Ln chronic toxicity to *D. magna*. Both La and Gd affected the reproduction of *D. magna* after 21 days of exposure in a different manner. La exposure resulted in a slight delay in the release of the first brood, while exposure to Gd decreased the total offspring of the organisms. These effects were not dependent on dissolved metal concentrations but appeared to be more closely related to Ln bioaccumulation and biodistribution within the organism. These results suggest that dietary exposure is an important uptake route under chronic exposure because the Ln bioaccumulated more strongly than through the waterborne route. The observed reproductive effects appear to occur when metals accumulate in the gut, which confirms the importance of taking the dietary exposure into account for long-term bioassays. Additional studies need to be conducted to explore the effects of Ln on the gut and energy limitations, aiming to gain a better understanding of the mode of action of these emerging contaminants on *D. magna*.

## Supplementary Information

Below is the link to the electronic supplementary material.

Supplementary Material 1
