# Supplementary material for: Toxicity and biodistribution of lanthanum and gadolinium in Daphnia magna following chronic dietary and waterborne exposure
Source: Ecotoxicology. 2026 Jan 3;35(2):27. doi: 10.1007/s10646-025-03013-4 (PMC12764634; doi:10.1007/s10646-025-03013-4)
Supplement: Supplementary file 1 — Supplementary Material 1 [file 10646_2025_3013_MOESM1_ESM.docx]

**S1:** Visual representation of the exposure conditions in the present study, showing the exposure to microalgae used as food in the chronic daphnid test and the four exposure conditions for the chronic daphnid test. ISO and M4 represent the different culture media used for the microalgae and daphnids, respectively.


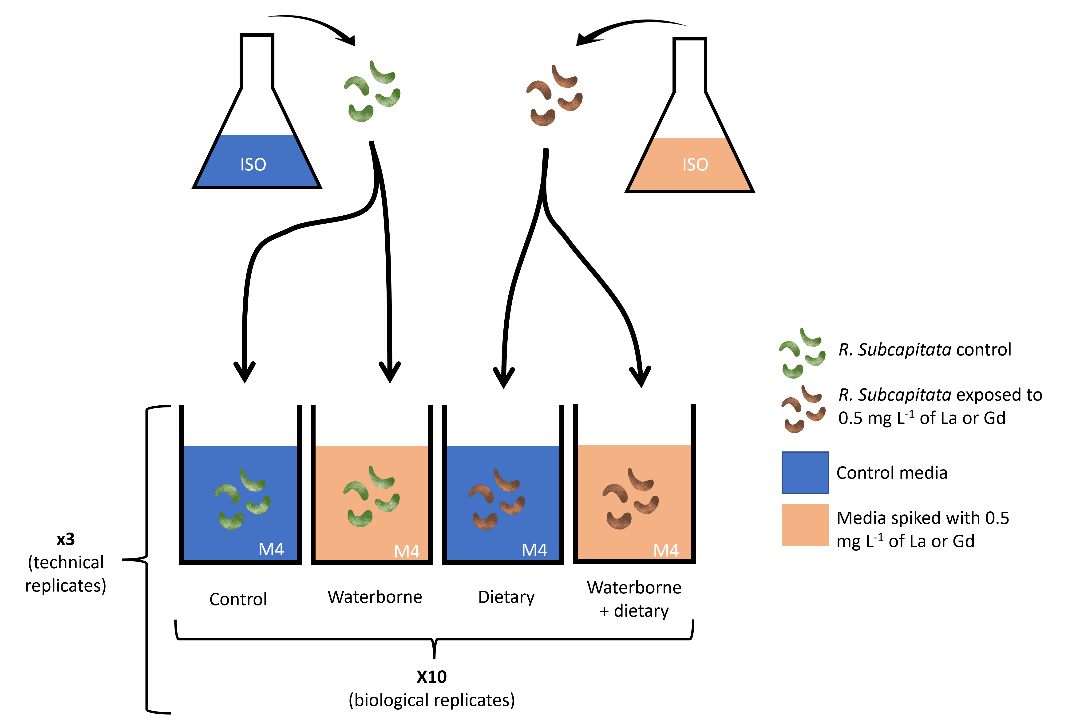


**S2:** Outcomes of statistical tests analysed with GraphPad Prism version 9.3.1 for Windows showing for all of the tests.

S2a: Kolmogorov-Smirnov tests

|  | La | | | |
| --- | --- | --- | --- | --- |
|  | Ctr | D | W | DW |
| Body burden in daphnids | <0.0001 | N too small | N too small | N too small |
| Body burden in algae | 0.0003 | - | N too small | - |
| Daphnid size | >0.10 | >0.10 | >0.10 | >0.10 |
| Daphnid weight | N too small | N too small | N too small | N too small |
| First brood release | <0.0001 | <0.0001 | 0.002 | 0.001 |
| Total neonates | >0.10 | >0.10 | >0.10 | 0.012 |

|  | Gd | | | |
| --- | --- | --- | --- | --- |
|  | Ctr | D | W | DW |
| Body burden in daphnids | 0.0001 | >0.10 | >0.10 | >0.10 |
| Body burden in algae | - | - | N too small | - |
| Daphnid size | <0.0001 | >0.100 | 0.091 | 0.091 |
| Daphnid weight | N too small | N too small | N too small | N too small |
| First brood release | 0.0001 | <0.0001 | <0.0001 | <0.0001 |
| Total neonates | 0.007 | 0.027 | 0.020 | 0.033 |

S2b: Multiple comparisons Kruskal-Wallis test for Ln accumulated per *R. subcapitata* cell (Figure 1)

|  | Adjusted P Value | Z |
| --- | --- | --- |
| Ctr vs. La | **0.005** | 3.17 |
| Ctr vs. Gd | **0.041** | 2.465 |
| La vs. Gd | >0.999 | 0.61 |

S2c: Multiple comparisons Kruskal-Wallis test for Ln content in *D. magna* after 21 days of exposure (Figure 2)

|  | La | | Gd | |
| --- | --- | --- | --- | --- |
|  | Adjusted P Value | Z | Adjusted P Value | Z |
| Ctr vs. D | **0.008** | 2.968 | **0.018** | 3.208 |
| Ctr vs. W | 0.651 | 2.551 | 0.064 | 1.605 |
| Ctr vs. DW | <0.0001 | 2.522 | **0.070** | 4.732 |
| D vs. W | 0.678 | 0.336 | >0.999 | 1.585 |
| D vs. DW | >0.999 | 0.000 | >0.999 | 1.247 |
| W vs. DW | **0.019** | 0.300 | >0.999 | 2.948 |

S2d: Kruskal-Wallis tests for Ln content in D. magna after 21 days of exposure (Figure 5)

|  |  | La |  |
| --- | --- | --- | --- |
|  |  | Mann-Whitney U | q value |
| Size | Ctr vs. D | 77.00 | >0.999 |
|  | Ctr vs. W | 78.00 | >0.999 |
|  | Ctr vs. DW | 45.00 | 0.866 |
| Weight | Ctr vs. D | 2.00 | >0.999 |
|  | Ctr vs. W | 2.00 | >0.999 |
|  | Ctr vs. DW | NA | NA |
| First brood release | Ctr vs. D | 139.50 | 0.152 |
|  | Ctr vs. W | 183.00 | 0.191 |
|  | Ctr vs. DW | 124.00 | **0.007** |
| Total nonates | Ctr vs. D | 207.50 | 0.788 |
|  | Ctr vs. W | 228.00 | 0.312 |
|  | Ctr vs. DW | 193.00 | 0.176 |

|  |  | Gd |  |
| --- | --- | --- | --- |
|  |  | Mann-Whitney U | Q value |
| Size | Ctr vs. D | 196.00 | 0.312 |
|  | Ctr vs. W | 186.00 | 0.299 |
|  | Ctr vs. DW | 174.00 | 0.116 |
| Weight | Ctr vs. D | 1.50 | 0.312 |
|  | Ctr vs. W | 3.00 | 0.707 |
|  | Ctr vs. DW | 4.50 | >0.999 |
| First brood release | Ctr vs. D | 313.00 | 0.467 |
|  | Ctr vs. W | 312.50 | 0.446 |
|  | Ctr vs. DW | 365.00 | 0.844 |
| Total nonates | Ctr vs. D | **242.50** | **0.017** |
|  | Ctr vs. W | 301.50 | 0.183 |
|  | Ctr vs. DW | 236.50 | 0.020 |

**S3:** La (right) and Gd (left) dissolved concentration (in logarithmic scale) under four exposure condition and three dose of microalgae. White: control, green (symbol /): dietary exposure, purple (symbol \): waterborne exposure, red (symbol ·): dietary + waterborne exposure. 3 replicates per conditions. Error bars: Standard deviations. The significant differences between the control and the different exposures are indicated by *p<0.05. **p<0.01. ***p<0.001

**S4:** Equations of each linear curves representing the algae consumption over time of the organisms exposed to La (right) and Gd (left). The slope of each linear curve represents the rate of algal cell ingested per organism per day.

|  | La | Gd |
| --- | --- | --- |
| Control | y = 1.5E6x + 6.3E6 | y = 1.4E6x + 1.7E7 |
| Dietary | y = 8.4E5x + 9.4E6 | y = 1.3E6x + 9.6E6 |
| Waterborne | y = 9.4E5x + 1.0E7 | y = 1.1E6x + 1.3E7 |
| Dietary + Waterborne | y = 1.3E6x + 1.2E6 | y = 1.1E6x + 1.4E7 |
